# BARRIERS AND FUTURE DIRECTIONS FOR SUFFICIENT ACADEMIC RESEARCH ON HOME MODIFICATION FOR OLDER ADULTS IN CHINA

**DOI:** 10.1093/geroni/igz038.936

**Published:** 2019-11-08

**Authors:** Mengzhao Yan

**Affiliations:** Leonard Davis School of Gerontology, University of Southern California, Los Angeles, California, United States

## Abstract

Home modification has been perceived as an effective method to make current dwelling units more age-friendly and improve the well-being of older adults. In China, a country with an increasing number of older adults, home modification research is still in its infancy. By typing “适老化改造” (home modification for older adults) as a keyword for the theme of articles in China National Knowledge Infrastructure (CNKI), the most authoritative and comprehensive database for academic publication in China, only 158 related papers are found to be published until the end of 2017, among which the first was published in 2009. With an aim to determine the current state of home modification research in older adults and to determine what could be done to facilitate sufficient research on this topic, the present study conducted a systematical review of the 158 papers about home modification for older adults published in China between 2009 and 2017. Based on the systematical review, the present paper identifies four barriers to conducting this line of research and puts forward six practical suggestions for future research studies in this area so as to contribute to building age-friendly dwellings and promoting age-friendly China.

